# Levels of C-Reactive Protein Associated with High and Very High Cardiovascular Risk Are Prevalent in Patients with Rheumatoid Arthritis

**DOI:** 10.1371/journal.pone.0006242

**Published:** 2009-07-16

**Authors:** Jonathan Graf, Rebecca Scherzer, Carl Grunfeld, John Imboden

**Affiliations:** 1 Department of Medicine, University of California San Francisco, San Francisco, California, United States of America; 2 Division of Rheumatology, San Francisco General Hospital, San Francisco, California, United States of America; 3 Division of Endocrinology and Metabolism, San Francisco VA Medical Center, San Francisco, California, United States of America; Lerner Research Institute, Cleveland Clinic, United States of America

## Abstract

**Objective:**

C-reactive protein (CRP) levels>3 mg/L and>10 mg/L are associated with high and very high cardiovascular risk, respectively, in the general population. Because rheumatoid arthritis (RA) confers excess cardiovascular mortality, we determined the prevalence of these CRP levels among RA patients stratified on the basis of their RA disease activity.

**Methods:**

We evaluated physician and patient global assessments of disease activity, tender and swollen 28 joint counts, erythrocyte sedimentation rate (ESR), and CRP measured in a single clinic visit for 151 RA patients. Disease activity was calculated using the Clinical Disease Activity Index (CDAI) and the Disease Activity Score 28 Joints (DAS28-ESR and DAS28-CRP).

**Results:**

Median CRP level was 5.3 mg/L. 68% of patients had CRP>3 mg/L, and 25% had CRP>10 mg/L. Of those with 0–1 swollen joints (n = 56), or 0–1 tender joints (n = 81), 64% and 67%, respectively, had CRP>3 mg/L, and 23% and 20%, respectively, had CRP>10 mg/L. Of those with remission or mildly active disease by CDAI (n = 58), DAS28-ESR (n = 39), or DAS28-CRP (n = 70), 49–66% had CRP>3 mg/L, and 10–14% had CRP>10 mg/L. Of patients with moderate disease activity by CDAI (n = 51), DAS28-ESR (n = 78), or DAS28-CRP (n = 66), 67–73% had CRP>3 mg/L, and 25–33% had CRP>10 mg/L.

**Conclusion:**

Even among RA patients whose disease is judged to be controlled by joint counts or standardized disease scores, a substantial proportion have CRP levels that are associated high or very high risk for future cardiovascular events in the general population.

## Introduction

Rheumatoid arthritis (RA) is a chronic inflammatory disease whose predominant clinical manifestations are synovitis and progressive articular damage. Patients with RA, however, experience excess cardiovascular morbidity and mortality that are not explained by Framingham cardiac risk factors but that have been linked to chronic systemic inflammation [1]–[7]. To date, C-reactive protein (CRP), a sensitive indicator of systemic inflammation, is the best biomarker for the excess cardiovascular disease associated with RA. Serum levels of CRP are independent predictors for preclinical cardiovascular disease (CVD), cardiovascular events, and overall cardiovascular mortality in RA patients [8]–[13]. CRP independently correlates with preclinical atherosclerotic disease in RA patients, as assessed by measurements of carotid intima media thickness, carotid plaque, coronary calcification, aortic pulse wave velocity, and endothelial cell dysfunction [10]–[15].

A substantial body of evidence also implicates systemic inflammation in the pathogenesis of atherosclerosis and CVD in the general population [16]. Elevated serum levels of CRP independently predict future cardiovascular events and preclinical CVD in the general population [17]–[21]. In a joint scientific statement, the American Heart Association (AHA) and Centers for Disease Control (CDC) categorized individuals whose CRP levels are>3 mg/L as being at high risk for future CVD events, citing their two-fold increased risk compared to those with a baseline CRP<1 mg/L [22]. However, the relationship between CRP and cardiovascular events is linear, not dichotomous [23]. Thus, individuals with CRP levels in the 1–3 mg/L range have an elevated risk of future cardiovascular events when compared to patients whose CRP is<0.5 mg/L [23]. At the other end of the spectrum, those with CRP>10 mg/L are at greater risk than those with CRP levels of 3 to 10 mg/L and are considered to be at very high risk of future cardiovascular events [23].

Serum CRP levels in RA patients frequently are above the 3 mg/L and 10 mg/L cutoffs associated with high and very high risk for CVD in the general population. For example, cross-sectional data from a recent observational cohort of 767 RA patients showed the median CRP level to be 11 mg/L, indicating that>50% of those RA patients had CRP levels associated with very high cardiovascular risk [24]. For comparison, less than 5% of individuals in the Women's Health Study had CRP levels>10 mg/L [17], [23].

Current therapeutic regimens can improve or suppress articular manifestations in many RA patients [25]. It is not known, however, whether control of articular disease is associated with effective suppression of the systemic inflammation that has been linked to excess CVD in RA. Accordingly, we performed a cross-sectional analysis of a cohort of RA patients in order to determine whether CRP levels that are associated with high or very high cardiovascular risk are prevalent in patients whose articular disease is controlled. Because there is no single uniformly accepted measure of RA disease activity, we judged successful control using each of the following: the physician global assessment of disease activity, the patient global assessment of disease activity, the number of swollen joints, the number of tender joints, and two standardized assessments of RA activity: the Clinical Disease Activity Index (CDAI) and the Disease Activity Score-28 joints (DAS28).

## Materials and Methods

### Patients

The 151 patients in this study were enrolled from the RA clinic at San Francisco General Hospital and are part of the University of California San Francisco (UCSF) RA cohort. All patients met ACR 1987 classification criteria for RA and had their therapeutic regimens determined by a UCSF-affiliated rheumatologist. For this study, we included all enrollees who had tender and swollen 28 joint counts, a patient global assessment of disease activity, erythrocyte sedimentation rate (ESR), and CRP determined during a single clinic visit between October, 2006 and April, 2008. Data were extracted from the most recent clinical encounter in which these criteria were met. 145 patients also had a physician's global assessment recorded at that encounter. This study was approved by the UCSF Committee on Human Research, and all patients signed consent documents allowing their clinical information to be gathered and analyzed for research purposes.

### Clinical Assessments

Data regarding patient age, gender, self-reported ethnicity, disease duration, seropositivity, medication use, and radiographic changes were extracted from each patient's clinical record. Four UCSF faculty rheumatologists trained in the DAS evaluation performed the tender and swollen joint counts and physician global assessment. Swollen and tender joint counts were analyzed categorically as: 0 joint, 1 joint, 2–4 joints, and 5+ joints. Patient and physician global assessments of disease activity were recorded independently using a standard 100 mm horizontal visual analog scale in which 0 = no activity and 100 = maximal activity [26]. The Clinical Disease Activity Index (CDAI), a validated measure of disease activity that does not incorporate biomarkers of inflammation, was calculated using swollen and tender joint counts and the physician and patient global assessments [24]. Because 6 patients did not have a physician global assessment recorded, 145 patients were analyzed using the CDAI. CDAI was categorized as remission (0–2.8) or mild (>2.8 to 10), moderate (>10 to 22), and severe activity (>22) using published cut points [27]. In contrast to the CDAI, the DAS28 quantifies RA disease activity using a formula that includes a measure of systemic inflammation, either ESR (DAS28-ESR) or CRP (DAS28-CRP), as well as the patient global assessment and the tender and swollen joint counts [28]. The DAS28-ESR and DAS28-CRP were analyzed using the published cut points for remission (≤2.6) or mild (>2.6 to 3.2), moderate (>3.2 to 5.1), and severe activity (>5.1) [29].

### Laboratory Measurements

All laboratory specimens were collected at the time of the clinic visit, with testing conducted in the San Francisco General Hospital clinical laboratories. ESR was measured according to standard Westergren techniques. High sensitivity CRP was measured in serum that had been frozen and stored at −20°C for less than 4 days. CRP assays were performed with a Beckman Coulter IMMAGE Nephelometry System (Fullerton, CA), using a near-infrared particle immunoassay, with a laser diode at 940 nm, a detection limit of 0.20 mg/L, and a measuring range of 0.20–1440 mg/Liter.

### Statistics

Spearman correlation coefficients were calculated to examine the associations of clinical characteristics, joint counts and CRP, since many measures were found to be non-normally distributed. The prevalence of elevated CRP was compared across ordered joint, DAS28, and CDAI categories using the Cochran-Armitage test for trend [30]. Elevated CRP levels were defined as>3 mg/L and 10 mg/L, levels associated with high and very high risk, respectively, of future cardiovascular events in the general population [23]. Median CRP levels were compared across categories using the non-parametric Jonckheere-Terpstra test [31]. All analyses were conducted using the SAS system, version 9.1 (SAS Institute, Inc., Cary, NC).

## Results

### Demographics and disease characteristics

The mean age of the patients was 52.4 years old, and the mean duration of RA was 9.9 years (Table 1). 89% of patients were female, a slightly higher prevalence of women than is typically reported, and, unlike most cohorts of RA in the United States, the majority was of Hispanic or Asian origin (Table 1). 84% and 82% of the study population were positive for rheumatoid factor and anti-cyclic citrullinated peptide (anti-CCP) antibodies, respectively (Table 1). 70% of patients had either joint space narrowing or erosive changes identified on radiographs of the hands or feet (Table 1).

**Table 1:** Subject Demographics and Clinical Characteristics.

Parameter	Statistic	
N		151
Age (y)	Median (IQR)	53.0 (45.0–62.0)
	Mean±SD	52.4±13.6
RA Disease Duration	Median (IQR)	7.9 (3.8–12.3)
	Mean±SD	9.9±8.5
Gender	Female	135 (89%)
Ethnicity	Asian/Pacific Islander	50 (33%)
	Black/African American	16 (11%)
	Latino/Hispanic	73 (48%)
	White/Caucasian	9 (6%)
	Other	3 (2%)
Rheumatoid Factor	n (%)	127 (84%)
Anti-CCP Positive	n (%)	118 (82%)
Radiographic Changes	n (%)	105 (70%)
Physician Global	Median (IQR)	27.0 (17.0–45.0)
Assessment	Mean±SD	31.2±19.4
Patient Global	Median (IQR)	47.0 (28.0–64.0)
Assessment	Mean±SD	47.4±22.7
Swollen Joint Count	Median (IQR)	2.0 (0.0–7.0)
	Mean±SD	4.7±5.6
Tender Joint Count	Median (IQR)	1.0 (0.0–4.0)
	Mean±SD	3.3±5.1
DAS28-ESR	Median (IQR)	3.9 (3.2–5.0)
	Mean±SD	4.1±1.4
DAS28-CRP	Median (IQR)	3.3 (2.6–4.3)
	Mean±SD	3.5±1.2
CDAI	Median (IQR)	12.5 (7.9–22.0)
	Mean±SD	15.8±11.4
ESR (mm/hr)	Median (IQR)	29.0 (14.0–45.0)
	Mean±SD	32.3±23.9
CRP (mg/L)	Median (IQR)	5.3 (2.2–9.9)
	Mean±SD	11.4±21.1
	n (%)>3 mg/L	103 (68%)
	n (%)>10 mg/L	37 (25%)
DMARD use	n (%)	124 (82%)
Biologic use	n (%)	41 (27%)

Anti-CCP, antibodies to cyclic citrullinated peptides.

DAS28, disease activity score 28 joints.

CDAI, clinical disease activity index.

DMARD, synthetic disease modifying antirheumatic drug.

### Disease activity in this patient population

The median physician global assessment of disease activity was 27, and the median patient global assessment was 47 (Table 1). The median numbers of swollen and tender joints in this population of RA patients were 2 and 1, respectively, out of a total possible joint count of 28 (Table 1). The median DAS28-ESR and DAS28-CRP were 3.9 and 3.3, respectively (Table 1). The median CDAI was 12.5 (Table 1). Median ESR was above normal (ESR 29 mm/h vs.≤20 mm/h). Median and mean CRP levels were 5.3 mg/L and 11.4 mg/L, respectively.

### CRP levels associated with high cardiovascular risk in patients with low disease activity as measured by physician or patient global assessments

**Figure 1:**
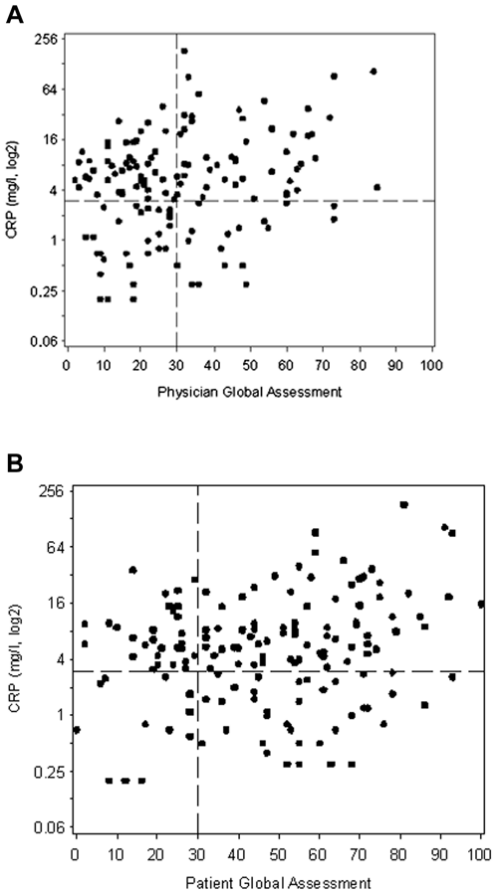
Scatterplots of CRP Levels by (a) Physician and (b) Patient Global Assessments of Disease Activity. CRP (log2) levels and global assessments were determined at the same clinic visit. The global assessments were recorded independently using a 100 mm visual analog scale in which 0 = no disease activity and 100 = maximal disease activity. The dotted reference lines denote a global assessment of 30 and CRP = 3 mg/L. CRP levels>3 mg/L are associated with high cardiovascular risk in the general population according to a scientific statement from the AHA and the CDC (7).

Sixty-eight percent of study patients had CRP levels>3 mg/L (Table 1). Because over two-thirds of our patients had CRP levels associated with high cardiovascular risk [22], [23], we examined CRP levels in relation to clinical assessments of disease activity. A majority of those patients who were judged by their physicians to have mild disease activity (defined as a physician global assessment of less than 30 out of 100) nonetheless had levels of CRP>3 mg/L (50 out of 79 = 63%). Indeed, the prevalence of CRP>3 mg/L was similar to that in the patients judged to have moderate or severe disease by physician global assessment of≥30 (46 out of 66 = 74%, p = 0.21), and the overall correlation of the physician global assessment of disease activity with CRP was weak (r = 0.20, p = 0.014, Figure 1a).

Likewise, a substantial majority of those patients with self-rated low disease activity (scores of less than 30 out of 100), had CRP>3 mg/L (29 out of 41 = 71%) which was similar to those with a patient global assessment of≥30 (74 out 110 = 67%, p = 0.84). The patient global assessment of disease activity had a weak, although still statistically significant, overall correlation with CRP (r = 0.19, p = 0.022, Figure 1b).

### CRP levels in patients with minimal clinical joint findings

**Figure 2:**
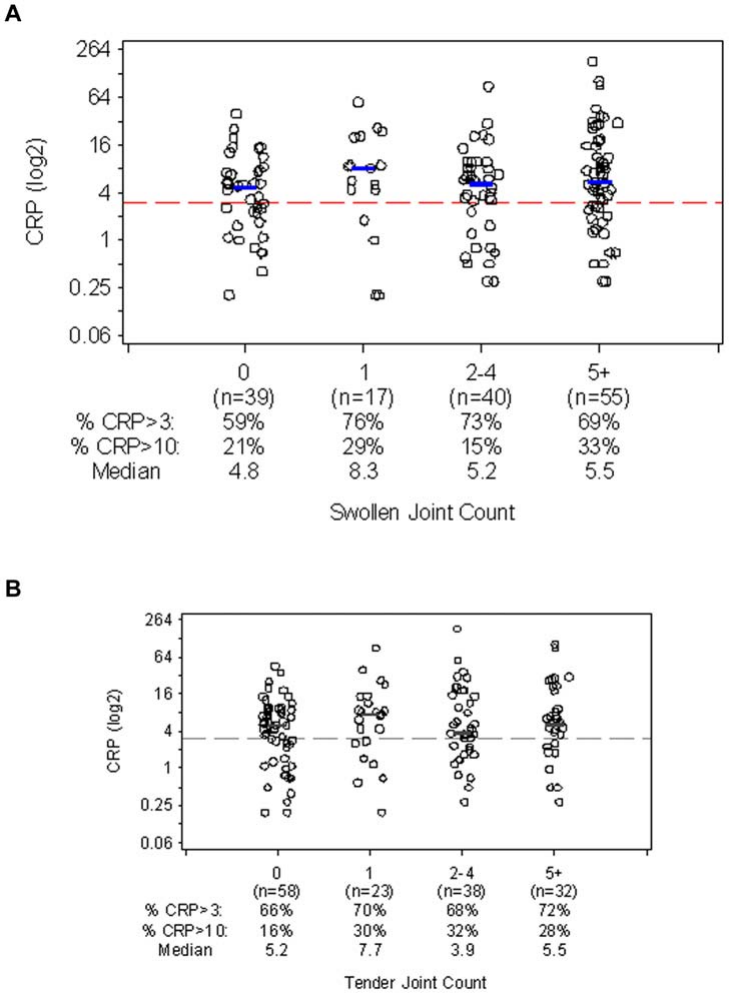
CRP Levels by (a) Swollen and (b) Tender Joint Count Category. CRP (log2) values are shown within each swollen joint count category. CRP levels and joint counts were determined at the same clinic visit. Blue lines denote location of the median. The dotted reference line is at CRP = 3 mg/L.

The low median number of affected joints (Table 1) indicated that a substantial proportion of patients in the study had little or no clinically detectable synovitis. Indeed, 39 patients had no swollen joints, while another 17 had only one swollen joint (Figure 2a). Despite little clinical evidence of articular disease in these two groups, 59% of those with no swollen joints, and 76% with only 1 swollen joint, had CRP levels>3 mg/L (Figure 2a). The prevalence of CRP>3 mg/L in those with 0, 1, 2–4, or 5+ swollen joints was similar (p = 0.38), and there was little trend toward increasing CRP levels (p = 0.19). CRP did not correlate significantly with the swollen joint count overall (r = 0.13, p = 0.12).

Similar results were observed when the tender joint count was used to assess articular disease. 66% of patients with no tender joints (n = 58) and 70% of those with 1 tender joint (n = 23) had CRP levels>3 mg/L (Figure 2b). The median CRP levels in these 2 groups were elevated at 5.2 and 7.7 mg/L, respectively. The prevalence of elevated CRP levels remained similar across all tender joint categories (p = 0.61 across all categories). CRP did not correlate significantly with the tender joint count overall (r = 0.11, p = 0.19), and there was little trend toward increasing median CRP levels (p = 0.28).

### Prevalence of elevated CRP levels in patients with disease activity assessed by CDAI

**Figure 3:**
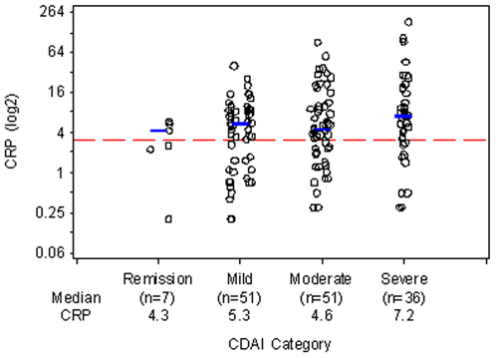
CRP Levels by Disease Activity as Determined by CDAI. CRP (log2) values are shown within each CDAI category. CRP levels and CDAI were determined at the same clinic visit. Blue lines denote location of median. The dotted reference line is at CRP = 3 mg/L.

Only 7 patients were in remission by CDAI, and, of these, 4 had CRP levels>3 mg/L (Figure 3). Of the 51 RA patients with mild disease activity by CDAI, 67% had CRP levels>3 mg/L, and the median CRP level in this group was 5.3 mg/L (Figure 3; Table 2). The prevalence of CRP>3 mg/dl was the same in those with moderate disease by CDAI (67%), and the median CRP was 4.6 mg/L (Figure 3; Table 2). Overall, CDAI showed a weak, but statistically significant positive correlation with CRP (r = 0.18, p = 0.027; Figure 3).

**Table 2:** Prevalence of CRP>3 mg/L and>10 mg/L in patients categorized by level of disease activity.

CRP Level	Measure	Remission	Mild	Moderate	Severe	P-value
CRP>3	CDAI	57%	67%	67%	75%	0.36
	DAS28-ESR	50%	47%	73%	79%	**0.0046**
	DAS28-CRP	42%	84%	70%	93%	**0.0011**
CRP>10	CDAI	0%	16%	25%	36%	**0.0091**
	DAS28-ESR	5%	16%	26%	38%	**0.0038**
	DAS28-CRP	5%	19%	33%	47%	**0.0001**

### CRP levels in patients with disease activity assessed by DAS28

**Figure 4:**
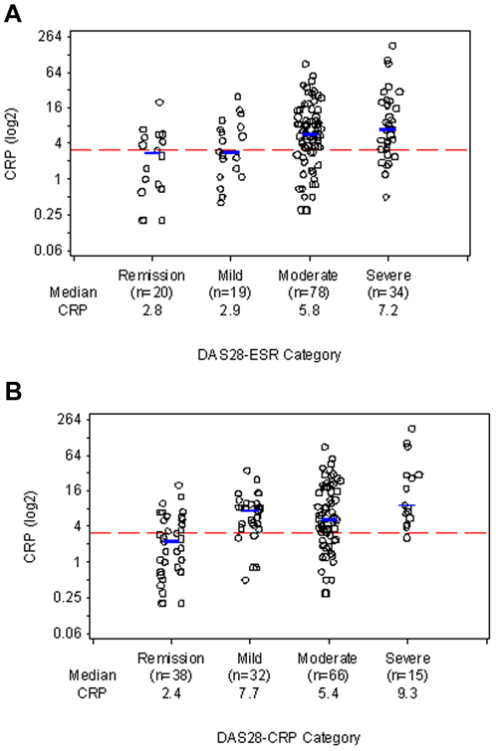
CRP Levels by Disease Activity as determined by (a) DAS28-ESR and (b) DAS28-CRP. CRP (log2) values are shown within each swollen joint count category. CRP levels and DAS28 were determined at the same clinic visit. Blue lines denote location of median. Dotted reference line is at CRP = 3 mg/L.

In our cohort of patients, those classified by DAS28-ESR as in remission or with mildly active disease had median CRP levels of 2.8 and 2.9 mg/L, respectively - lower than the levels of CRP found in patients similarly classified by CDAI (Figure 4a). However, a substantial percentage of patients in remission or with mildly active disease by DAS28-ESR (50% and 47% respectively) still had CRP levels>3 mg/L (Figure 4a; Table 2). Of those with moderate disease activity by DAS28-ESR (n = 78), 73% had CRP levels>3 mg/L (Figure 4a; Table 2). When the DAS28-CRP was used instead to classify disease activity, more patients were categorized as having remission or mild disease; 42% of patients in remission, 84% of patients with mild activity, and 70% of those with moderate activity had CRP levels>3 mg/L (Figure 4b; Table 2).

### Prevalence of CRP levels associated with very high cardiovascular risk

Overall, 25% of our RA patients had CRP levels>10 mg/L (Table 1). Of patients thought to have well-controlled disease by their physicians (physician global assessment scores<30) or by themselves (patient global assessment scores<30), 15% and 20%, respectively, had CRP>10 mg/L (Figure 1). When disease was assessed by joint counts, 21% of patients with 0 swollen joints had CRP>10 mg/L, as did 29% of patients with 1 swollen joint (Figure 2a). Similarly, 16% of patients with no tender joints and 30% with 1 tender joint had CRP>10 mg/L (Figure 2b).

Among patients who met criteria for remission, the prevalence of CRP>10 mg/L ranged for 0% for CDAI to 5% for DAS28-ESR and DAS28-CRP (Table 2). However, a substantial proportion of patients with mild disease activity by CDAI (16%), DAS28-ESR (16%), or DAS28-CRP (19%) had CRP levels>10 mg/L (Table 2). The percentages were higher still in those with moderate disease activity by these measures: 25–33% (Table 2).

## Discussion

Our data demonstrate that a substantial proportion of RA patients thought to have suppressed disease nonetheless have CRP levels that are associated with high (>3 mg/L) and very high (>10 mg/L) risk of cardiovascular events in the general population. Our findings should be viewed in the context of currently recommended therapeutic targets in RA. For example, the American College of Rheumatology (ACR) recommends a therapeutic goal of remission or mild disease activity by CDAI or DAS28 [32]. In our study, 47% to 83% of those with mild activity by these measures had CRP levels>3 mg/L, and 16% to 19% had CRP levels>10 mg/L. In its guidance on the use of TNF inhibitors for RA, the United Kingdom's National Institute for Health and Clinical Excellence accepts moderate disease activity and requires severe activity by DAS28 for the introduction of TNF inhibitors [33]. We observed that 70% to 73% of our patients with moderate disease activity by DAS28 criteria had CRP levels>3 mg/L and that 26% to 33% of these had CRP levels>10 mg/L.

The basis for the high prevalence of elevated CRP levels in RA patients with mild to moderate disease activity is not certain but likely is multifactorial. First, the clinical joint exam lacks sensitivity to detect subtle synovitis and may underestimate the extent of active rheumatoid disease. Indeed, magnetic resonance imaging can detect inflammation in rheumatoid joints clinically thought to be free of synovitis [34]. Second, chronic inflammation at extraarticular sites (e.g., gingivitis) might stimulate CRP production. None of our patients had clinically apparent infections at the time of analysis, and CRP levels in our patients with mild disease activity are stable over time (unpublished observations), arguing against an intercurrent event as a source of elevated CRP. Third, the low prevalence of smoking in the SFGH RA cohort (12.5%) excludes an important contribution of smoking to CRP levels in our study. Finally, adipose tissue, particularly visceral fat, is a source of CRP production and can make contributions to the serum CRP levels of RA patients independent of their disease activity [35].

Several studies suggest that treatment of RA with methotrexate or TNF inhibitors may reduce cardiovascular events and mortality due to CVD [36]–[39], but the improvement may only occur in those who have a response to therapy [39].

The great majority of patients with moderate disease activity or better in our study were on methotrexate or other synthetic disease-modifying anti-rheumatic drugs. Among the patients receiving TNF inhibitors in our study, the median CRP level (6.0 mg/L) and the prevalence of CRP>3 mg/L (76%) or>10 mg/L (27% ) did not differ significantly from those of patients not using TNF inhibitors, but the patients receiving these agents appeared to have more severe disease (data not shown).

While therapies may reduce some of the overall cardiovascular risk of a population of RA patients, there are limited data on the extent of residual cardiovascular risk in individual treated RA patients. For example, it remains to be seen whether treating RA patients to levels of mild disease activity or remission, as assessed by the best available clinical metrics, normalizes cardiovascular risk relative to that of the general population. However, our finding that a majority of patients who achieve these ACR-recommended targets nonetheless have elevated CRP levels suggests that excess cardiovascular risk persists. Consistent with this possibility, endothelial dysfunction was reported in a study of young to middle aged patients with RA who were in remission or had mild disease activity by DAS28-ESR and who were free from other cardiovascular risk factors. In that study, endothelial dysfunction correlated with average CRP levels and disease duration [10].

Our study has several limitations. First, it is cross-sectional. However, studies demonstrating CRP to be an independent predictor of cardiovascular risk, including those involving RA patients, often used a single, baseline measurement of CRP [9], [17]–[19], [23]. Second, we examine a biomarker for cardiovascular risk (CRP) rather than cardiovascular outcomes. Levels of cardiovascular risk have not been assigned to specific values of CRP in RA, as has been done in the general population. However, the available evidence, although limited in scope, does not support the notion of differential cardiovascular risk for a given level of CRP between the general population and patients with RA. For example, in a 10 year observational study of newly diagnosed RA, patients whose baseline CRP levels were≥5 mg/L had a hazard ratio of death from CVD of 14.7 (95% CI 2.0–109.2) relative to those with baseline CRP levels<4 mg/L [9]. Moreover, RA patients with even modestly elevated CRP levels (>1.92 mg/L) are more insulin-resistant than those with lower CRP levels [40]. Inflammation-induced insulin resistance contributes to the deleterious effects of inflammation on the cardiovascular system [41].

Our study indicates that systemic inflammation, as reflected in an elevated CRP, persists in a sizable number of RA patients with minimal or no clinically detectable joint disease and thus may confer increased cardiovascular risk upon these patients. Future studies should examine the relation of subclinical joint inflammation detected by sensitive imaging techniques to markers of cardiovascular risk. If this risk predicted here is confirmed by future studies, then recognition of these at-risk RA patients may have important implications for therapy. One option might be to base treatment decisions on disease assessment scores more heavily weighted towards markers of inflammation or to treat subclinical joint inflammation. Given the efficacy, toxicity, and expense of current RA therapies, however, an alternative strategy might be to aggressively modify traditional cardiovascular risk factors in those with persistent systemic inflammation. Even in the absence of elevated low density lipoprotein, the use of statins might reduce cardiovascular risk in RA patients with elevated CRP. Of note in this regard, the recently published JUPITER trial of apparently healthy individuals without hyperlipidemia but with CRP≥2 mg/L demonstrated that statin therapy significantly reduced major cardiovascular events, with a hazard reduction of 0.56 (p<0.00001) [42]. With respect to RA, our results point to the need to define the extent to which the persistent systemic inflammation places individual RA patients at risk for CVD and to determine the cardiovascular benefits of more aggressive RA therapy, of risk modification, and of the use of statins.
